# Gender specific differences in left ventricular remodelling in obesity may explain differences in obesity related mortality

**DOI:** 10.1186/1532-429X-14-S1-P75

**Published:** 2012-02-01

**Authors:** Oliver J Rider, Adam J Lewandowski, Richard Nethononda, Steffen E Petersen, Alex Pitcher, Cameron Holloway, Rajarshi Banerjee, James P Byrne, Paul Leeson, Stefan Neubauer

**Affiliations:** 1 Cardiovascular Medicine, University Of Oxford, Oxford, UK; 2 Upper GI Surgery, Southampton General Hospital, Southampton, UK; 3 Physiology Anatomy and Genetics, University Of Oxford, Oxford, UK

## Summary

Across both genders, obesity, in the absence of traditional cardiovascular risk factors, is characterized by concentric LV remodeling. Whereas males show predominantly concentric remodeling, females exhibit eccentric and concentric hypertrophy. This may explain the observed gender difference in obesity related cardiovascular mortality.

## Background

Obesity related cardiovascular mortality, although elevated when compared to normal weight, is lower in females (♀) than males (♂) at every body mass index (BMI) level. Given the fact that males have less fat tissue than females at each BMI level, the reasons behind this trend are unlikely to be attributable to the effects of excess adiposity alone.

As different patterns of left ventricular (LV) remodeling have been shown to have varying prognostic value, with concentric hypertrophy being more strongly predictive of cardiovascular mortality than eccentric hypertrophy, our aim was to investigate whether sex-specific differences in LV remodeling could provide an additional explanation for the observed gender difference in obesity related mortality.

## Methods

703 subjects (♀ n= 390, ♂ n=313) without identifiable cardiovascular risk factors, (BMI range 15.7-59.2 Kg/M2) underwent cardiovascular magnetic resonance at 1.5 Tesla for the assessment of LV mass (g), end-diastolic volume (EDV; ml) and LV mass/volume ratio (LVM/VR).

## Results

**Figure 1:**
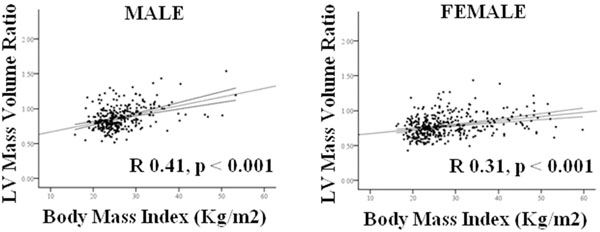
Gender differences in relationship between BMI and LV mass/volume ratio.

**Figure 2:**
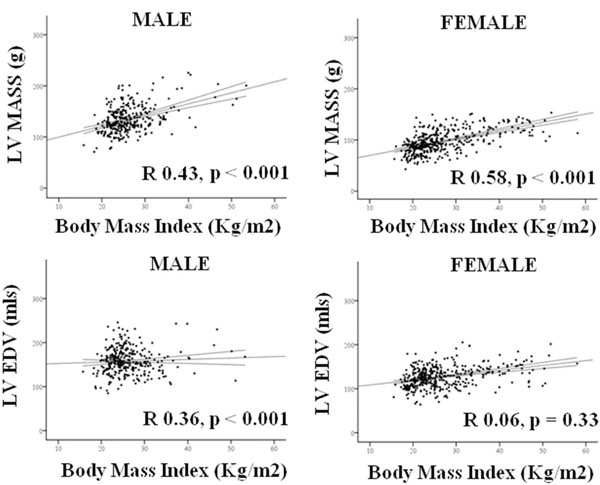
Gender differences in relationship between BMI and LV mass (upper panels) and LV EDV (lower panels).

Although concentric remodeling was present in both sexes, with LVM/VR being positively correlated to BMI (♂ R 0.41, ♀ R 0.31, both p<0.001). On linear regression analysis the degree of concentric hypertrophy was greater in males, with a steeper regression coefficient being observed (♂ +0.13 vs ♀ +0.06 LVM/VR increase per BMI point increase, p=0.0001, Figure 1). Whereas males showed a greater LV hypertrophic response to increasing BMI (LV mass increase; ♂ +2.2g vs ♀ +1.6g per BMI point increase, p=0.001), females showed a greater LV cavity dilatation response (♀ +1.1mls vs ♂ +0.31mls per BMI point increase, p<0.001). Indeed, in contrast to females, where BMI and LV-EDV were positively correlated (R.37, p<0.001) BMI was not correlated with EDV in men (R0.06, p=0.33, Figure 2).

## Conclusions

Across both genders, obesity, in the absence of traditional cardiovascular risk factors, is characterized by concentric LV hypertrophy. Whereas males show predominantly concentric remodeling, females also exhibit a degree of cavity dilatation suggesting that a mixed pattern of eccentric and concentric hypertrophy is present. Given the fact that concentric hypertrophy is a more powerful predictor of cardiovascular risk, this may go some way to explain the observed gender difference in obesity related cardiovascular mortality.

## Funding

The study was supported by a grant from the Wellcome Trust and by the Oxford Partnership Comprehensive Biomedical Research Centre with funding from the Department of Health's NIHR Biomedical Research Centres funding scheme. SN acknowledges support from the Oxford BHF Centre of Research Excellence.

